# The Link between Gut Dysbiosis Caused by a High-Fat Diet and Hearing Loss

**DOI:** 10.3390/ijms222413177

**Published:** 2021-12-07

**Authors:** Dagmara Kociszewska, Jeffrey Chan, Peter R. Thorne, Srdjan M. Vlajkovic

**Affiliations:** Department of Physiology and The Eisdell Moore Centre, Faculty of Medical and Health Sciences, The University of Auckland, Private Bag, Auckland 1142, New Zealand; d.kociszewska@auckland.ac.nz (D.K.); jeffrey.chan@auckland.ac.nz (J.C.); pr.thorne@auckland.ac.nz (P.R.T.)

**Keywords:** gastrointestinal tract, inner ear, high-fat diet, obesity, microbiota, dysbiosis, hearing loss

## Abstract

This review aims to provide a conceptual and theoretical overview of the association between gut dysbiosis and hearing loss. Hearing loss is a global health issue; the World Health Organisation (WHO) estimates that 2.5 billion people will be living with some degree of hearing loss by 2050. The aetiology of sensorineural hearing loss (SNHL) is complex and multifactorial, arising from congenital and acquired causes. Recent evidence suggests that impaired gut health may also be a risk factor for SNHL. Inflammatory bowel disease (IBD), type 2 diabetes, diet-induced obesity (DIO), and high-fat diet (HFD) all show links to hearing loss. Previous studies have shown that a HFD can result in microangiopathy, impaired insulin signalling, and oxidative stress in the inner ear. A HFD can also induce pathological shifts in gut microbiota and affect intestinal barrier (IB) integrity, leading to a leaky gut. A leaky gut can result in chronic systemic inflammation, which may affect extraintestinal organs. Here, we postulate that changes in gut microbiota resulting from a chronic HFD and DIO may cause a systemic inflammatory response that can compromise the permeability of the blood–labyrinth barrier (BLB) in the inner ear, thus inducing cochlear inflammation and hearing deficits.

## 1. Introduction

Hearing is essential for quality of life, cognitive and socioemotional development, and general health at all stages of life. In adults, hearing loss is associated with cognitive decline [1] and represents a significant risk factor for the development of dementia [2]. Over 1.5 billion people worldwide experience some decline in hearing ability during their lifetime, of whom at least 430 million will be affected by disabling hearing loss [3]. The World Health Organisation (WHO) estimates that by 2050, nearly 2.5 billion people will be living with some degree of hearing loss, of whom at least 700 million will require rehabilitation services [3]. Hearing loss is also a substantial economic issue; the WHO estimates that the overall global cost of unaddressed hearing loss is more than USD 980 billion annually [4].

In the last few years, epidemiological studies have suggested that a high body mass index (BMI) in the obesity range, and to a lesser extent, in the overweight range, is positively associated with hearing loss [5,6,7,8,9,10,11]. In addition, a diet high in cholesterol is associated with an increased risk of developing sensorineural hearing loss (SNHL) [12,13]. On the other hand, treatment with statins and consumption of monounsaturated fats may reduce the risk of SNHL [14,15]. To date, cochlear microangiopathy, impaired insulin signalling, oxidative stress (OS) and dyslipidaemia were implicated as prospective mechanisms linking a high-fat diet (HFD) to hearing loss [12,16,17,18,19]. As obesity continues to be a significant public health issue worldwide, further studies are necessary to establish whether early interventions and lifestyle changes could reduce the risk of hearing loss. There is also a need to understand how obesity increases the risk for hearing loss, such as via inflammation, genetic, endocrinological, or a combination of these factors. This review was undertaken to further investigate the links between the gut and inner ear, which we provisionally named the *gut–inner ear axis*. Based on clinical cases and animal studies, this review discusses the association between gut health and inner ear function and the link between gut dysbiosis and SNHL. A comprehensive summary of the existing literature regarding the proposed gut–inner ear axis hypothesis is provided in this review.

A literature search of related publications from 1995 to 2021 was conducted using PubMed, Google Scholar and Embase medical databases. In addition, reference lists from the relevant papers were used. Following Boolean search logic, the main keywords included: (hearing loss OR SNHL OR sensorineural hearing loss OR cochlea OR microangiopathy) and (diet OR obesity OR diabetes OR diet-induced obesity OR inflammation) and (microbiota OR gut dysbiosis OR reactive oxidative species OR lipopolysaccharide OR LPS OR high-fat diet OR intestinal hyperpermeability OR inflammatory bowel disorder). The results of the search were consequently examined according to their relevance to this review. Only English language publications were included.

## 2. Gut Microbiota

The gastrointestinal (GI) tract protects the body from environmental challenges by blocking access to the host’s blood circulation for many pathogens, including fungi, bacteria, and parasites. This GI firewall comprises various structures and shielding mechanisms to fulfil its protective function, constituting an intestinal barrier (IB). The IB includes mucosal and submucosal layers, tight junctions (TJ), continuously renewing epithelium, and microbiota [20,21,22,23]. The intestinal epithelial cells (IECs) are a barrier between the immune system and GI lumen, maintaining a limited IB permeability [24]. Nonetheless, the GI tract has an arduous task. The constantly changing environment requires the IB to continually respond to new challenges, yet maintain its integrity while protecting the host from pathogens [20]. Failure of the IB leads to intestinal hyperpermeability (leaky gut), allowing pathogens and their metabolites to enter the bloodstream [25].

One of the principal challenges the IB faces relates to diet. An unhealthy diet can weaken the GI firewall, allowing toxic or undesired substances to penetrate the mucosal and submucosal layers, and seep into the bloodstream, which leads to activation of the immune system. Failed GI homeostasis can lead to immunoinflammatory responses in the gut and cause conditions such as irritable bowel syndrome, celiac disease, inflammatory bowel disease (IBD), or spread through the bloodstream to other organs, disrupting their function [20,26].

Intestinal (gut) microbiota can be considered a first line of defence against GI pathogens, as it stimulates the immune system, synthesises amino acids and vitamins, and breaks down toxic compounds found in food [27]. The human microbiome contains 3.8 × 10^13^ microorganisms, including thousands of various species of bacteria, fungi, parasites, and viruses [28], accounting for up to 3% of body weight [29]. The majority of the microbiota inhabit the distal bowel, where they help process materials otherwise indigestible for humans [28]. Each person has a distinctive and unique microbiome [30], and alterations in the gut microbiota can benefit or harm the host [20,31]. The microbiome can react dynamically in response to changing conditions such as diet or medications. When the balance between the different microorganisms is disturbed, it leads to dysbiosis, affecting the regular, necessary interactions between the body and microbiota and altering the IB, thus making the host’s body more prone to illness. When this barrier is compromised, the host immune system can initiate a cascade of inflammatory responses to the intestinal milieu, turning the symbiotic relationship between the host and gut microbiome into a pathological one [32]. The most frequent sources of dysbiosis are antibiotics, bacteria-depleting medications, infectious diseases, or prolonged, poor diet [33].

### 2.1. Gut Microbiota and Inflammation

In eubiosis (healthy microbiome), the gut is protected by the outer mucus layer, anti-inflammatory microbial products and elements of the immune system, such as immunoglobulin A (IgA), regulatory T cells (Tregs), and eosinophils. The human microbiome consists of the five main phyla of bacteria: *Firmicutes, Bacteroidetes, Proteobacteria, Actinobacteria, Verrucomicrobia* and *Archaea*, with the most dominant being *Bacteroides, Prevotella*, and *Ruminococcus* [34].

Bacteria can have pro- and anti-inflammatory properties. As an example of an anti-inflammatory bacterium, *Akkermansiamuciniphila* is a mucin-degrading strain belonging to the phylum *Verrucomicrobia.* In eubiosis, *Akkermansiamuciniphila* enhances mucin production [35], and supports the expression of TJ proteins such as occludin, thus maintaining IB integrity [36]. It is also associated with leanness, reduced inflammation and insulin sensitivity [37,38,39,40,41].

In contrast, pro-inflammatory bacteria produce endotoxins. In dysbiosis, the increase in Gram-negative and lipopolysaccharide (LPS)-producing bacteria can be observed [42,43]. It is well established that a HFD is closely linked with DIO-related dysbiosis, which manifests in a decrease in overall microbiota, and increased gut permeability [44,45]. Several studies using murine and human models have associated DIO and a HFD with enhanced endotoxemia, which leads to the increased permeability of the IB and increased penetration of the luminal LPS into the circulation [46,47,48,49,50].

Therefore, intestinal hyperpermeability can cause a leak of toxic substances (such as pro-inflammatory microbial solutes) into the bloodstream [25]. In response to pathogens spreading through the bloodstream, the body launches an acute inflammatory response by activating the innate and adaptive immune systems [51]. If the inflammatory response fails to resolve, it will lead to a state of chronic inflammation that could potentially reach the inner ear. Moreover, this chronic inflammation can result in complications such as diabetes or IBD [23].

IBD is an umbrella term used to describe disorders that involve chronic GI inflammation, such as Crohn’s disease (CD) and ulcerative colitis (UC) [52], which are also characterised by a leaky gut. The prevalence of genetic causation of IBD is relatively low, making environmental factors a key suspect. Moreover, factors that are known to positively impact gut microbiota, such as early exposure to animals, having many siblings [53,54], natural mode of delivery [55] and breastfeeding [56], decrease the chances of developing IBD. In contrast, factors such as C-section [55,57], excessive paediatric hygiene [58] and early-life antibiotic therapies [59,60,61] appear to increase the risk of developing IBD. This is further supported by the incidence of IBD being higher in urban areas rather than rural areas [62,63]. Diet is also an important factor, where multiple studies have described the association between diet and the incidence of IBD [64,65,66,67,68,69].

Although the complex aetiology of IBD is not fully understood, IBD and sub-clinical manifestations of inflammatory gut diseases have been associated with gut dysbiosis and significantly increased levels of bacterial products such as LPS [46,70,71]. Previous studies have shown that healthy subjects can generally tolerate autologous microbiome; however, in susceptible individuals, the breakdown of this symbiosis is associated with chronic intestinal inflammation [72,73]. Treatments that typically affect microbiota, such as faecal diversion and antibiotic therapy, are often used for IBD management [74,75,76,77,78,79]. Interestingly, the intestinal lesions in IBD are usually located at sites of higher concentrations of commensal bacteria [80,81]. Moreover, IBD patients have an altered gut mucus layer [82], which would further implicate microbiota in the pathophysiology of IBD. IBD patients also present higher yields of antibodies against commensal bacteria than healthy individuals [83].

In eubiosis, *Bacteroides fragilis* is a commensal, anti-inflammatory bacterium of the gut. Many strains of *B. fragilis* produce polysaccharide A (PSA), which binds to TLR2, promoting secretion of the anti-inflammatory cytokine interleukin 10 (IL-10) from the regulatory T cells. Outside the gut (due to surgery or leaky gut), *B. fragilis* can cause systemic inflammation. A particular *B. fragilis* strain (ETBF) produces metalloprotease enterotoxin, which is thought to be involved in the aetiology of IBD [84,85,86].

### 2.2. Lipopolysaccharides

Lipopolysaccharides (LPS) are components of the cellular wall of Gram-negative bacteria. Under physiological conditions, the IB prevents bacterial LPS from entering the bloodstream [87]. Pathological conditions can increase the number of Gram-negative bacteria in the GI tract [46] and the levels of LPS circulating in the bloodstream, leading to metabolic endotoxemia [88], as shown in mice on a HFD [47]. The increased levels of LPS are also found in metabolic diseases and DIO [22,46,47,89,90]. LPS activates inflammatory responses after binding to Toll-like receptor 4 (TLR4) on the immune cells [89]. LPS also induces nuclear factor kappa-light-chain-enhancer of activated B cells (NF-κB) transcription factor in macrophages and stimulates these cells to release inflammatory cytokines such as interleukin 1 beta (IL-1β) and tumour necrosis factor alpha (TNFα) [91]. In turn, TNFα can suppress scavenger receptor function in macrophages [92]. In addition, LPS can increase the production of intracellular reactive oxygen species (ROS) [93].

Resulting from the disbalance of ROS metabolism, OS is recognised as a mediator of inflammation-induced disorders, including age-related hearing loss (ARHL) and other forms of SNHL [94,95,96,97,98]. In human studies, administration of LPS resulted in suppression of insulin receptors and increased insulin resistance [99], which has been associated with hearing loss [17].

### 2.3. Short-Chain Fatty Acids

Commensal, anti-inflammatory gut microbiota metabolises complex carbohydrates into short-chain fatty acids (SCFA). SCFA fulfil various roles, including activating and inhibiting the inflammatory response and different metabolic pathways [100]. SCFA levels vary between obese and lean individuals [101]. Butyrate, a type of SCFA, contributes to the prevention of metabolic disorders [102] and helps retain the IB’s integrity, thus reducing the rate of LPS translocation through the intestinal epithelium [102]. SCFA can also prevent HFD-induced obesity in mice by altering gut microbiota [103]. Furthermore, butyrate stimulates nuclear transcription factor-peroxisome proliferator-activated receptor gamma (PPAR-γ), which in turn inhibits the pro-inflammatory NF-κB pathway [104,105]. It also inhibits interferon-gamma (IFN-γ) signalling to suppress inflammation [106].

## 3. How Can Diet Affect the Intestinal Barrier?

Intriguingly, an increased prevalence of metabolic diseases has been observed in industrialised countries, where the so-called “Western diet” is dominant. The “Western diet” is characterised as a HFD, with a significant amount of refined carbohydrates, food additives and low fibre intake [107]. This diet is prevalent in low- and middle-income countries, where energy-dense food is chosen based on its lower price and higher accessibility. However, the Western diet is minimal in nutrients and lacks wholegrains, fruits, vegetables, and fibre [108]. Many authors have reported a correlation between the “Western diet” and a range of disorders that result in the hyperpermeability of the IB, such as IBD [52,57,67,109].

Interestingly, the severity of IBD correlates with the amount of fat in the diet [110], and the increased frequency of IBD flare-ups results from increased mucosal penetration by microbial antigens [110,111]. Increased LPS levels have been found in the plasma of IBD patients [112], providing evidence for intestinal hyperpermeability leading to a systemic inflammatory response. Indeed, IBD often presents with several extraintestinal manifestations (EIMs), which may result from chronic inflammation. These comorbidities include arthritis, psoriasis, uveitis, cardiovascular disease, neuropsychological disorders, and metabolic syndromes, to name a few [113]. SNHL has also been acknowledged as an EIM associated with IBD and celiac disease [114,115,116]. However, to date, very little research has addressed this correlation.

### High-Fat Diet and Intestinal Permeability

It is still unclear how a HFD can cause IB hyperpermeability and systemic inflammatory processes that result in life-changing disorders. One of the most discussed mechanisms is that the expression and distribution of intestinal apical junctional complexes (AJC) are affected by a HFD, leading to IB hyperpermeability [26], as demonstrated in mice [47,117,118]. Another possibility is that a HFD significantly increases bile acid production and secretion from the gall bladder. The bile acid emulsifies luminal fat and exposes micelles to lipase-mediated digestion, thus damaging the mucosal lining of the IB [26,119,120,121].

However, there is an unmistakable relationship between a HFD, DIO, and inflammation. Murine studies have shown that gut microbiota was altered in animals fed a HFD, with an increased number of Gram-negative bacteria that produce LPS [47]. Recent evidence suggests that HFD triggers intestinal hyperpermeability via LPS turn-over mechanisms [26]. LPS can cause intestinal hyperpermeability via directly modulating TJ organisation, stimulating Toll-like receptor 4-cluster of differentiation 14 (TLR4-CD14)-mediated activation of NFκB, and inducing IEC dysfunction [122,123]. The structural part of LPS-Lipid A, comprising branched-chain short fatty acids, is a ligand site for the pro-inflammatory TLR4-CD14 signalling complex within IECs and immune cells [26,124]. LPS can also be incorporated in the chylomicrons, which transport dietary lipids from the intestines via lipid A tail, and are distributed into the bloodstream, causing systemic inflammatory responses [125]. Thus, dietary fats can increase serum LPS and cause endotoxemia [47,126]. In clinical studies, people consuming a low-fat diet had serum LPS levels between 0 and 0.2 ng/mL, whereas the group that consumed a HFD had up to 2 ng/mL of serum LPS [127].

Several animal studies have shown that a HFD affects key cytokines and inflammatory pathways. Pro-inflammatory mediators such as IL-1β, interleukin 6 (IL-6), and TNF-α can affect IEC signalling, viability, and cohesiveness, resulting in IB hyperpermeability [26,128,129,130,131,132,133]. Mice on a HFD show reduced interleukin 7 (IL-7) levels responsible for TJ organisation [134]. Within a week of consuming a HFD, intestinal anti-inflammatory cytokine interleukin 10 (IL-10) levels decreased in rats [135,136]. Mice on a HFD also show reduced interleukin 22 (IL-22) levels, the cytokine that enhances the wound healing response and decreases fatty-acid induced endoplasmic reticulum (ER) stress [133,137].

Furthermore, dietary fats disturb the epithelial shedding–proliferation axis. In response to a hostile environment, IECs shed every two to six days and are replaced by new enterocytes, guided by Wnt signalling, and a proliferative layer located on the base of Lieberkühn’s crypts [138,139]. This process is highly organised, sophisticated, and susceptible to multiple stimuli [140]. The IECs of the intestinal lumen are coated with a superficial unstirred mucus layer (SUML), consisting of antimicrobials, immunoglobulin A (IgA), bicarbonate, glycoproteins, and lubricant. The SUML protects the enterocytes by filtering toxins, bacteria, and insoluble substances [141,142]. HFD-induced inflammation favours enterocyte apoptosis, which results in structural “gaps” within the basement membrane, resulting in the B hyperpermeability [143,144], followed by extraintestinal endotoxemia [145,146].

## 4. Interactions between the Gut and Distant Organs

Gut microbiota communicates with the brain through several communication channels known as the microbiota–gut–brain axis. The communication routes include tryptophan metabolism, the vagus nerve, microbial metabolites, and the immune system [147]. Even though the notion of a gut–brain axis is relatively new, the evidence is exponentially rising [21,147,148,149,150,151,152].

Interaction between microbiota and the host’s tissues within the IB can result in the secretion of chemokines, cytokines, neurotransmitters, endocrine messengers, neuropeptides, and microbial by-products such as LPS [147]. These molecules can then penetrate the vascular and lymphatic systems, impact neural messages carried out by vagal and spinal afferent neurons, communicate with the brain regarding the host’s health status and influence behaviour [147]. Most of the host–microbiota interactions occur within the IB, where the exchange of molecules mediates communication between the gut and immune system [153]. The intestinal epithelium also houses enterocytes, secretory cells, chemosensory cells and gut-associated lymphoid tissue, participating in the immune response [154].

The enterocytes play a vital role in the innate immune system response by releasing pro- and anti-inflammatory cytokines and chemokines, whereas B lymphocytes in Peyer’s patches produce immunoglobulins [155]. Intestinal epithelial pattern recognition receptors (PRRs) identify molecular patterns unique to the specific microorganisms [156,157]. These include Toll-like receptor (TLR) family members, which mediate pro-inflammatory responses [158]. In addition, some metabolites of commensal bacteria (such as LPS) can activate the innate immune system and, thus, modulate neural responses [147,159].

The integrity of the blood–brain barrier (BBB) can be affected by systemic pro-inflammatory signalling induced by a HFD [160,161,162], leading to brain inflammation and injury. This may not be linked to obesity, as transplantation of microbiota from mice fed a HFD caused significant disruptions in exploratory, cognitive, and stereotypical behaviour in non-obese recipient mice fed a standard diet [163]. This study provided direct evidence that HFD-induced changes to the gut microbiome are sufficient to disrupt brain function in the absence of obesity. Furthermore, previous studies have shown that germ-free mice exhibit increased BBB permeability, with downregulation of TJ proteins claudin-5 and occludin. Nevertheless, exposure of germ-free mice to specific-pathogen-free mice followed a reduction in BBB permeability and upregulation of TJ proteins [164].

The gut microbiota can also modulate the host’s behaviour and brain function [149,165,166,167,168]. Of interest, germ-free mice displayed cognitive deficits, similar to mice infected with *Citrobacter rodentium* and exposed to acute stress [169,170]. Several studies have reported gut microbiota as a critical player in neuroinflammation, neurodegeneration and mental illness development [171,172]. Gut dysbiosis has been said to play a role in the aetiology of neurodegenerative diseases such as Alzheimer’s disease (AD) [173], Parkinson’s disease (PD) [174], multiple sclerosis [175] and amyotrophic lateral sclerosis [176]. In AD, an imbalance in the microbiome is associated with the co-localisation of LPS with amyloid β-protein (Aβ) 1–40/42 in amyloid plaques and around blood vessels [177]. LPS binds to microglial receptors (TLR2, TLR4 and CD14), activating the NF-κB (p50/p60) complex, which initiates neuroinflammatory processes [178,179]. 

Regarding PD, bowel inflammation can provoke the progression of the disease [180]. Colonic hyperpermeability in PD patients has been linked with increased α-synuclein and *E. coli* accumulation in the sigmoid colon [181]. The microbiome landscape in PD is significantly shifted, with decreased levels of bacteria that produce anti-inflammatory mediator butyrate (*Blautia*, *Coprococcus*, and *Roseburia*) and increased levels of LPS-producing bacteria (*Oscillospira* and *Bacteroides*) [174]. Furthermore, specific changes in the gut microbiome have been linked with the severity of symptoms and elevated serum cytokine levels seen in PD [182]. This further supports a link between changes in gut microbiota, systemic inflammation and PD.

IBD has also been linked with a higher incidence of neurodegenerative disorders, including dementia [183,184]; however, the mechanisms are not well understood.

In summary, factors such as a HFD can cause gut dysbiosis, hyperpermeability of the IB and infiltration of pathogens and microbial solutes into the circulation, thus contributing to chronic systemic inflammation [26]. Chronic inflammation can disrupt the BBB’s integrity, facilitating the infiltration of pathogens and pro-inflammatory cytokines into the brain, resulting in neuroinflammation and neurodegeneration [185,186,187,188].

## 5. Could There Be a Similar Connection to the Inner Ear?

The central tenet of this review is that, in a dysbiotic state, metabolites and pro-inflammatory molecules from gut microbiota can pass through the blood–labyrinth barrier (BLB). In contrast, in a eubiotic state, the BLB protects the cochlea from pathogens, which would otherwise compromise cochlear integrity, causing inflammation and injury. This can be postulated based on evidence that the BBB [189,190,191] and the BLB [192,193] increase their permeability in response to the increased presence of the bacterial metabolite LPS.

### 5.1. The Blood–Labyrinth Barrier

The inner ear (comprising the auditory and vestibular peripheral organs) bears a resemblance to the brain in some respects (e.g., resident innate immune system, BLB that resembles BBB), making it comparably susceptible to influences from gut microbiota.

The cochlea contains an intricate vascular system that maintains cellular metabolism and the endocochlear potential as a driving force for sensory transduction [194]. Antigens, toxins, metabolites, and circulating immune cells from the periphery, if unrestricted, could infiltrate the cochlear blood supply [193,194]. Under normal circumstances, the BLB limits the infiltration of these molecules into the cochlea and, thus, plays a vital role in maintaining cochlear homeostasis and fluid dynamics [193,195], akin to the BBB [196,197].

**Figure 1:**
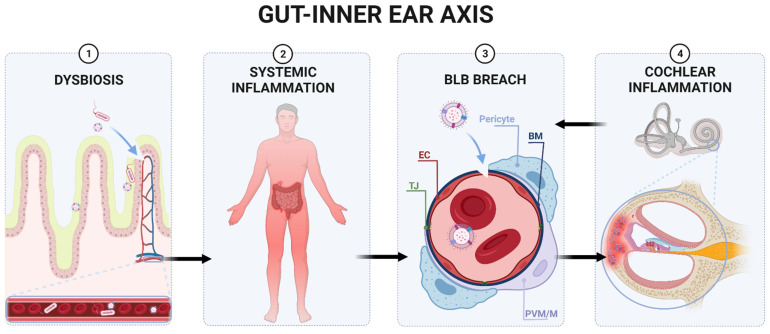
Proposed gut–inner ear axis resulting in sensorineural hearing loss (SNHL). (**1**) Gut dysbiosis induced by a high-fat diet can damage the intestinal barrier and cause a leaky gut. (**2**) This allows gut microbiota and bacterial toxins such as lipopolysaccharide to infiltrate the bloodstream and cause a systemic inflammatory response. (**3**) Pathogens and inflammatory cytokines reaching the inner ear can damage the blood–labyrinth barrier (BLB). (**4**) Infiltration of pathogens to the inner ear leads to the activation of resident macrophages, the release of pro-inflammatory cytokines, and the overproduction of reactive oxygen species (ROS), causing apoptosis of damaged cells and immune cell infiltration in the lateral wall of the cochlea (spiral ligament and stria vascularis). Inflammatory processes in the cochlea might further increase the BLB’s permeability, perpetuating inflammation. Unresolved inflammation leads to damage of sensorineural structures, eventually causing SNHL. Abbreviations: EC, endothelial cell; TJ, tight junction; BM, basement membrane; PVM/M, perivascular-resident macrophage-like melanocytes.

The stria vascularis (SV) and spiral ligament (SL) in the lateral cochlear wall are regions where the BLB is relatively well studied [194,195,198]. In these regions, vascular endothelial cells, held together by TJ, constitute the barrier’s first line of support, as the TJ proteins limit the permeability of solutes into the inner ear [198]. Pericytes and perivascular resident macrophages (PVM) represent the second line of support [194]. Pericytes of the SV and SL are rich in desmin fibres, which maintain the integrity of the vessels and regulate the expression of TJ proteins [199]. Cochlear PVM support local tissue homeostasis, scavenge foreign proteins and mediate the inflammatory response to tissue injury [200] (Figure 1).

### 5.2. Cochlear Inflammation

Inflammation is a self-limiting physiological process with the primary goal of protecting the tissue from further injury and infection. However, the inability to resolve inflammation might cause further tissue damage; hence, inflammation can be regarded as both a friend and foe. Unresolved, chronic low-grade inflammation underlies the aetiology of many diseases. The recent English Longitudinal Study of Ageing has demonstrated a relationship between systematic inflammation and ARHL [201]. In this study, the low-grade secretion of pro-inflammatory cytokines, including TNF-α, IL-6, and IL-1β, has been postulated to mediate age-related cellular degeneration [201]. However, there is still an evident gap in understanding the link between chronic inflammation and SNHL.

Acute inflammation in the cochlea has two consecutive stages mediated by immune cells and molecules: induction and resolution. At the induction stage, the immune cells, pro-inflammatory cytokines, and ROS infiltrate the affected and adjacent tissues [202]. The innate immune system responds first, inducing ROS and cytokine production by macrophages which, in turn, attract more macrophages and neutrophils to kill and phagocytose the apoptotic cells. If the inflammation does not resolve, the adaptive immune system activates T-lymphocytes [202]. In sterile and pathogen-induced inflammation, stress signals mediate the cochlear inflammatory response via the NF-κB signalling pathway [203,204,205]. These stress signals include cytokines, chemokines, pro-inflammatory enzymes, and adhesion molecules [205]. Moreover, the damage to the cochlear sensory hair cells can lead to a release of ligands that interact with immune response-sensing receptors, such as TLRs [205].

### 5.3. Innate Cochlear Immunity and BLB

Cochlear innate immunity is essential in mediating bacterial and sterile inflammation as part of an integrated defence system against pathogens and exogenous stressors. Inflammation is initiated by pathogen recognition receptors that recognise and bind pathogen-associated molecular patterns and damage-associated patterns [206]. The activation of these receptors leads to the upregulation of pro-inflammatory cytokines, which recruit resident and circulating leukocytes to the site of inflammation [206]. These leukocytes are involved in the phagocytosis of cellular debris and foreign antigens and apoptosis of damaged cellular structures within the cochlea [207]. The BLB limits the extravasation of infiltrating leukocytes from the circulation to reduce cochlear damage from inflammation [194]. However, recent evidence suggests that BLB dysfunction can also occur during cochlear inflammation, which impacts the ability of the BLB to limit the entry of immune cells and inflammatory or infectious agents into the cochlea, which can exacerbate cochlear damage [192,194,208]. Similar mechanisms have been implicated in the pathogenesis of almost all major causes of acquired hearing loss, including acoustic trauma, ototoxicity, and age-related hearing loss [194,209,210]. Dhukhwa et al. [211] showed increased cochlear expression of NADPH oxidase 3 (*NOX3*), transient receptor potential vanilloid 1 (*TRPV1*) and inflammatory mediators TNF-α, inducible nitric oxide synthase (*iNOS*), and Cyclooxygenase-2 (*COX2*) in rats 48 h after noise exposure. However, expression of *TRPV1* and *TNF*-α was significantly higher at day 21 than after 48 h, indicating chronic, unresolved inflammation, which leads to cell damage and apoptosis in the cochlea and hearing loss. Moreover, chronic activation of the TRPV1 channel leads to intracellular Ca^2+^ accumulation in cochlear structures expressing these channels [212]. Ca^2+^ overload and ROS are associated with apoptosis and increased inflammatory response in many tissues [213,214].

SNHL could result from the failure to resolve inflammatory responses in the cochlea, leading to chronic cochlear inflammation and extensive damage to the sensory and neural structures (sensory hair cells and primary auditory neurons). Nevertheless, it is unclear whether systemic inflammation can directly cause SNHL or only intensify cell damage after an acute insult. Koo et al. showed that aminoglycoside-induced hearing loss could be exacerbated via increased vascular permeability resulting from LPS-induced endotoxemia [215]. In their study, a low-dose LPS significantly increased the concentration of ototoxic aminoglycosides in the cochlea and caused cochlear vasodilation, increasing levels of systemic and cochlear inflammatory markers. Furthermore, Yang and colleagues [216] demonstrated that virus-induced inflammation via Toll-like receptors TLR7 and TLR9 might potentiate the ototoxic effects of aminoglycoside therapy. The synergistic action of TLR7 and TLR9 agonists to stimulate the cochlear inflammatory response and kanamycin in mice resulted in impairment of outer hair cell function and cell death, infiltration of pro-inflammatory cytokines and Iba1+ macrophages into the cochlea and auditory brainstem response (ABR) threshold shifts. However, when applied without kanamycin, agonists of TLR7 and TLR9 did not affect ABR thresholds [211].

## 6. The Gut–Inner Ear Axis: Clinical and Experimental Evidence

Large-scale longitudinal studies have provided growing evidence for a chronic HFD as a risk factor for hearing loss due to its association with DIO and metabolic disease [7,10,11,215,217]. For example, Scinicariello and colleagues [10] found that the prevalence of high-frequency hearing loss in obese adolescents was significantly higher compared to normal-weight adolescents [10]. A cross-sectional study by Hwang et al. [215] was one of the first studies to point out a possible link between SNHL and central obesity in a group of 690 females and males between 35 and 85 years old. It was supported by a prospective cohort study [7], which demonstrated that a high BMI and obesity increase the risk for SNHL. Another retrospective cross-sectional study showed that childhood DIO could be correlated with higher hearing thresholds across all frequencies and an almost two-fold increase in unilateral low-frequency SNHL [216]. In addition, the increased total cholesterol and triglyceride levels and higher BMI were linked to a higher occurrence of sudden onset SNHL, most likely due to restricted blood supply to the cochlea [218].

Circumstantial evidence suggests that patients with IBD are more often and more significantly affected by SNHL than healthy controls [114,219,220]. Similarly, paediatric patients with celiac disease are more likely to develop SNHL than healthy children [115,116]. It was even suggested that children presenting with SNHL should be routinely checked for celiac disease [115].

### Diet-Induced Obesity (DIO) and Hearing Loss

**Figure 2:**
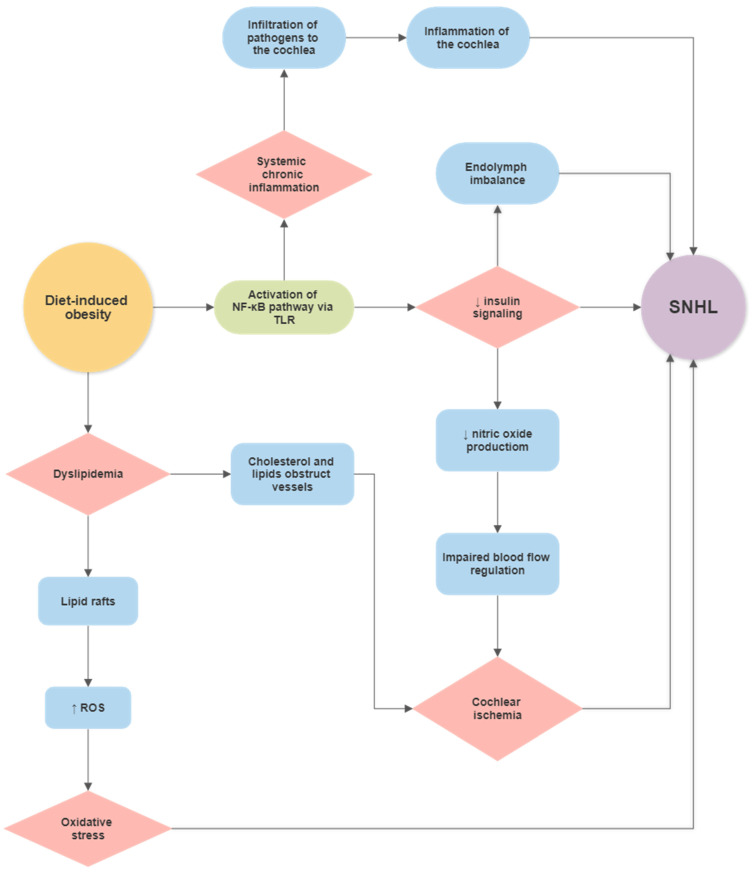
Flowchart showing the potential link between a high-fat diet and sensorineural hearing loss (SNHL) based on current literature.

Previous studies have identified cochlear microangiopathy, dyslipidaemia, redox imbalances, and changes in insulin signalling as potential mechanisms by which diet, obesity, and metabolic diseases can cause cochlear pathology and hearing deficits [5,215,221] (Figure 2). This section will delve further into possible pathophysiological mechanisms by which diet, DIO, and metabolic disease can become risk factors for hearing loss.

In the current literature, microangiopathic changes, such as the thickening of the vessel walls in the SV, have been identified in models of DIO and metabolic disease [8,222]. For instance, Hwang et al. demonstrated that obese mice had cochlear blood vessels in the SV with reduced diameters and thicker walls [8].

Subsequent atrophy of the SV may also result from these microangiopathic changes caused by metabolic disease in humans [222]. In mice, the fibrocytes of the SL and spiral ganglion neurons were prone to degeneration in response to DIO [8]. Hwang et al. [8] demonstrated that the expression levels of hypoxia-induced factor 1 (HIF-1), TNF-α, NF-κB, caspase 3, poly (ADP-ribose) polymerase-1, and apoptosis-inducing factor were significantly higher in the spiral ganglion and SL of obese mice. They also demonstrated that cell densities in the spiral ganglion and SL at the basal turn of the cochlea decreased as a result of DIO [8]. There is also a large body of evidence implicating DIO as a primary risk factor in developing insulin resistance and the clinical manifestation of insulin resistance, type 2 diabetes mellitus [223,224,225,226]. Subsequently, chronic systemic inflammation resulting from a HFD has been identified as a critical mechanism underlying decreased insulin signalling [18,227], obesity [228], neurodegeneration [229,230] and hearing loss [8,231,232]. The activation of pro-inflammatory pathways, particularly the NF-κB pathway, has been linked to reduced insulin signalling [18]. Insulin signalling in the cochlea contributes to the upregulation of the Na-K-Cl co-transporter 1 (NKCC1), which is crucial for the maintenance of the electrochemical composition of endolymph [233]. Due to its high potassium concentration, endolymph provides an electrochemical gradient required for the depolarisation of sensory hair cells and sensory transduction. Pålbrink and colleagues identified a reduction in insulin signalling in the cochlea and subsequent expansion of the endolymphatic compartment in mice on a chronic HFD [233]. They proposed that insulin resistance stemming from a chronic HFD results in reduced NKCC1 expression in the cochlea, which disrupts ionic homeostasis in the endolymph (Figure 2). Additionally, insulin resistance can reduce nitric oxide production in endothelial cells [234,235]. As nitric oxide is the principal regulator of cochlear blood flow [236,237], insulin resistance may reduce auditory vascular perfusion, leading to ischemia and damage to sensorineural and secretory tissues in the cochlea.

Dyslipidaemia is a condition characterised by altered levels of lipids, including triglycerides, cholesterol, and phospholipids [238]. Hearing loss likely results from an accumulation of cholesterol and lipid rafts in cochlear blood vessels [12]. Lipid rafts may interact with the ROS-generating nicotinamide adenine dinucleotide phosphate (NADPH) oxidases in the endothelial cell membrane to induce ROS overproduction [239,240]. Du et al. [232] investigated the effects of HFD-induced hyperlipidaemia in the cochlea of aged rats. They identified a significant increase in the expression of NADPH oxidases and ROS generation in hyperlipidaemic animals compared to rats on the control diet. ROS accumulation was also associated with expanding mitochondrial DNA mutations in the cochlea and mitochondrial dysfunction due to oxidative damage, which initiates cellular apoptosis in cochlear tissues [8,232].

## 7. Summary

Obesity and hearing loss are prevalent health problems globally. More than 1.5 billion people will experience some decline in hearing ability during their lifetime, of whom at least 430 million will be affected by disabling hearing loss. If not identified and addressed in a timely way, hearing loss can severely reduce the quality of life at various stages of life by delaying language development, reducing social engagement, and compromising economic independence and educational opportunities. Significantly, even mild levels of hearing loss increase the long-term risk of cognitive decline and dementia. Implementing appropriate strategies focussing on modifiable risk factors may reduce the overall burden of disease. One of those modifiable risk factors for hearing loss is low-grade systemic inflammation associated with a high-fat diet and obesity. This review proposes the existence of the gut–inner ear axis, which represents a novel approach to understanding the mechanisms regulating cochlear function in health and disease.
